# Identification and characterization of seven new exon 11-associated splice variants of the rat mu opioid receptor gene, OPRM1

**DOI:** 10.1186/1744-8069-7-9

**Published:** 2011-01-21

**Authors:** Jin Xu, Mingming Xu, Grace C Rossi, Gavril W Pasternak, Ying-Xian Pan

**Affiliations:** 1 Department of Neurology and Program in Molecular Pharmacology and Chemistry, Memorial Sloan-Kettering Cancer Center, New York, NY 10065, USA; 2 Department of Psychology, CW Post College, Long Island University, Brookville, NY 11568, USA

## Abstract

**Background:**

The mouse mu opioid receptor (OPRM1) gene undergoes extensive alternative splicing at both the 3'- and 5'-ends of the gene. Previously, several C-terminal variants generated through 3' splicing have been identified in the rat OPRM1 gene. In both mice and humans 5' splicing generates a number of exon 11-containing variants. Studies in an exon 11 knockout mouse suggest the functional importance of these exon 11-associated variants in mediating the analgesic actions of a subset of mu opioids, including morphine-6β-glucuronide (M6G) and heroin, but not others such as morphine and methadone. We now have examined 5' splicing in the rat.

**Results:**

The current studies identified in the rat a homologous exon 11 and seven exon 11-associated variants, suggesting conservation of exon 11 and its associated variants among mouse, rat and human. RT-PCR revealed marked differences in the expression of these variants across several brain regions, implying region-specific mRNA processing of the exon 11-associated variants. Of the seven rat exon 11-associated variants, four encoded the identical protein as found in rMOR-1, two predicted 6 TM variants, and one, rMOR-1H2, generated a novel N-terminal variant in which a stretch of an additional 50 amino acids was present at the N-terminus of the previously established rMOR-1 sequence. When expressed in CHO cells, the presence of the additional 50 amino acids in rMOR-1H2 significantly altered agonist-induced G protein activation with little effect on opioid binding.

**Conclusion:**

The identification of the rat exon 11 and its associated variants further demonstrated conservation of 5' splicing in OPRM1 genes among rodents and humans. The functional relevance of these exon 11 associated variants was suggested by the region-specific expression of their mRNAs and the influence of the N-terminal sequence on agonist-induced G protein coupling in the novel N-terminal variant, rMOR-1H2. The importance of the exon 11-associated variants in mice in M6G and heroin analgesia revealed in the exon 11 knockout mouse implies that these analogous rat variants may also play similar roles in rat. The complexity created by alternative splicing of the rat OPRM1 gene may provide important insights of understanding the diverse responses to the various mu opioids seen in rats.

## Background

Three families of opioid receptors were proposed from pharmacological studies [1,2]. Of the three opioid receptor families, the mu opioid receptors are particularly important since they mediate the actions of most of the clinically relevant opioids, as well as those most widely abused such as heroin. Clinicians have observed a wide range of responses among patients, a variability confirmed among different strains of mice. These findings, along with receptor binding studies and the actions of selective antagonists, led us to propose the existence of multiple mu opioid receptor subtypes [3] long before the molecular characteristics of mu receptors were known.

The molecular cloning of the mu opioid receptor (MOR-1) [4-6] opened new opportunities to investigate the molecular underpinnings for the concept of multiple mu opioid receptors. A single mu opioid receptor gene (OPRM1) has been identified in mammals, raising questions on how to reconcile a single gene with the multiple pharmacologically defined mu opioid receptors. One possibility is alternative pre-mRNA splicing, which can provide enormous RNA and protein diversity. A number of G protein-coupled receptors undergo alternative splicing, such as dopamine D_2 _[7,8], somatostatin 2 [9], prostaglandin EP_3 _[10], serotonin receptor subtypes [11], tachykinin NK(2) [12], metabotropic glutamate receptor [13,14], and metabotropic muscarinic acetylcholine receptors [15,16]. Antisense mapping studies provided early evidence suggesting alternative splicing of the mouse and rat OPRM genes [17,18], which was further supported by the studies in an exon 1 knockout (KO) mouse model generated by Pintar and colleagues [19]. In this mouse, loss of exon 1 eliminated all the full length variants, which contain exon 1. However, a series of exon 11-associated variants lacking exon 1 were still expressed. Pharmacologically, disrupting exon 1 in this mouse completely abolished morphine analgesia, but not that of either M6G or heroin, consistent with the possibility that alternatively spliced transcripts lacking exon 1 might be responsible for the residual M6G and heroin actions.

In recent years, alternative splicing of the OPRM1 genes has been extensively explored by our group and others [20-34]. In the mouse OPRM1 gene, over 28 alternatively spliced variants have been isolated. Of these splice variants, most are C-terminal variants that were generated through alternative splicing between exon 3 and 10 different downstream exons [35]. These C-terminal variants from mice, rats and humans bound mu opioids with similar high affinities, but displayed marked differences in agonist-induced G protein coupling in both their potency, defined by the EC_50 _values, and efficacy, indicated by the maximal stimulation[25,27,36,37]. Although it can be speculated that different C-terminal tails may alter interactions of receptor with different G proteins or other related proteins like regulator of G protein signaling (RGS) proteins based upon their intracellular location, the underlying mechanisms for these differences remain unclear. Morphine-induced internalization also varied among the C-terminal variants. For example, morphine given intracerebroventricularly in vivo internalized mMOR-1C in the mouse lateral septum, while mMOR-1 is not internalized by morphine [38]. Mu agonist-induced interaction of phosphorylated mu opioid receptor with β-arrestins have been indicated to involve the receptor internalization and desensitization [39-41]. Various additional phosphorylation sites for β-adrenergic receptor kinase, protein kinase C, caseine kinase, tyrosine kinase and cAMP- and cGMP-dependent protein kinases have been predicted among different C-termini [35]. Although highly speculative, these phosphorylation sites at different C-termini may differentially modulate the recruitment of β-arrestins and therefore contribute to the disparities in mu agonist-induced receptor internalization.

In the mouse, OPRM1 generates a set of splice variants associated with exon 11, located approximately 30 kb upstream of exon 1, under the control of a distinct exon 11 promoter [24,28]. Of nine exon 11-associated variants, three variants encoded the original mMOR-1 protein, five variants lacked exon 1 and predicted a 6 TM receptor protein, and one variant predicted a protein with single TM. The functional relevance of exon 11-associated variants was established by studies in an exon 11 KO mouse model [42]. Unlike the exon 1 KO mouse developed by Pintar [19], the exon 11 KO mouse retained full sensitivity towards morphine and methadone analgesia while the effects of M6G, fentanyl and heroin were greatly attenuated. This suggested that exon 11 associated variants mediated the actions of a subset of mu opioids, including M6G and heroin.

**Figure 1:**
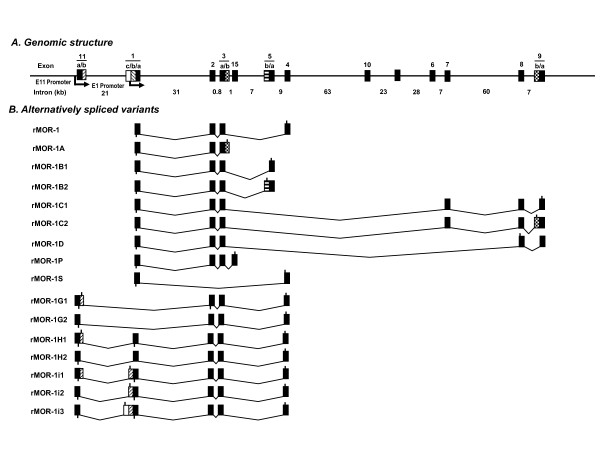
**Schematic of the rat OPRM1 gene structure and alternative splicing. **A. Genomic structure of the rat OPRM1gene. Exons and introns are showed by boxes and horizontal lines, respectively. Translational start and termination sites are indicated by downward and upward lines on exon boxes, respectively. Exons are numbered based upon their time of discovery, as previously reported. B. Alternatively spliced variants of the rat OPRM1 gene. Exon composition for each alternatively spliced variant was indicated by appropriate exon boxes. The lines between exons are introns that are spliced out during splicing. Translation start and stop points are shown by bars below and above exon boxes, respectively.

**Figure 2:**
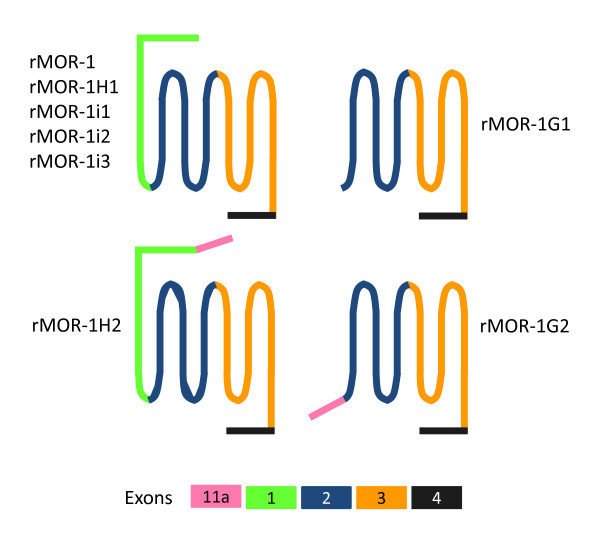
**Schematic of protein structures predicted from Exon 11-associated variants. **Colored bars indicate proteins predicted from different exons.

Previous studies also have reported many splice variants from the rat OPRM1 gene [20,25] (Figures 1 &2), as well as the human OPRM1 gene [21,26,28,32,33]. The current study reports the identification and characterization of the rat exon 11 homolog and seven exon 11-associated variants.

## Results

### Cloning the rat exon 11-associated splice variants

**Figure 3:**
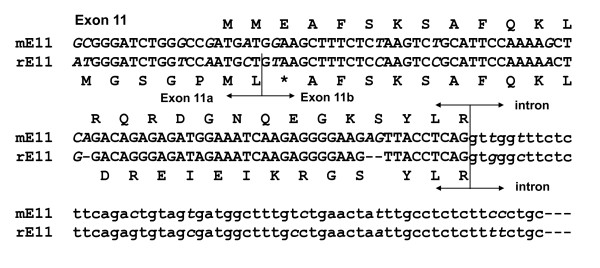
**Sequence comparison of the rat exon 11 with the mouse exon 11. **The nucleotide sequences and their deduced amino acids of the mouse exon 11 (mE11) and the rat exon 11 (rE11) are shown by capital letters. Intron sequences are indicated by low letters. The identical nucleotides are indicated by non-italic letters and diverse nucleotides by italic letters. Exon-exon and exon-intron boundaries are indicated by arrows.

**Figure 4:**
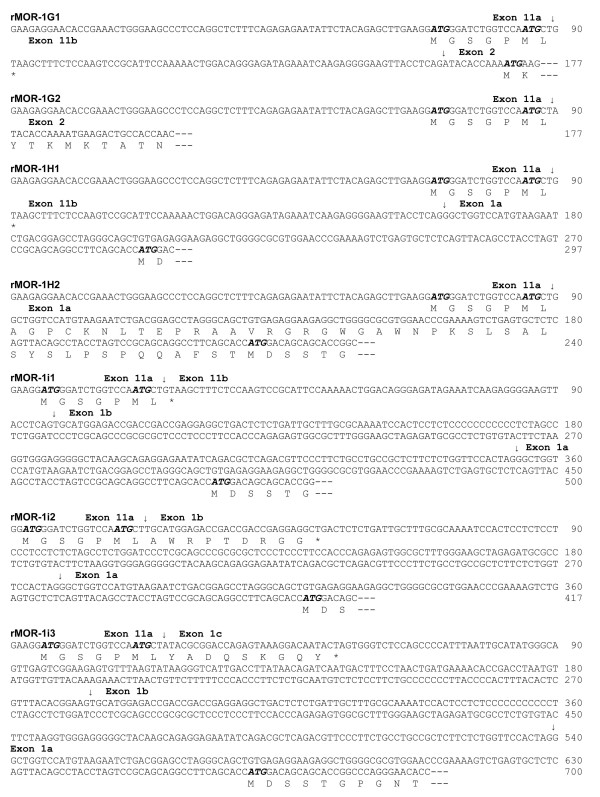
**The partial nucleotide sequence and predicted amino acid sequence of the rat variants. **Exon-exon boundaries are indicated by arrows. The stop codons are showed by *. The complete cDNA and deduced amino acid sequences of rMOR-1G1, rMOR-1G2, rMOR-1H1, rMOR-1H2, rMOR-1i1, rMOR-1i2 and rMOR-1i3 have been deposited in the GenBank database with Accession numbers: DQ680043, EU024650, EU340244, EU024651, EU340245, EU024652 and EU340246, respectively.

To determine whether the rat OPRM1 gene contained an exon homologous to the mouse exon 11, we blasted the rat genome database in Ensembl using the mouse exon 11 sequence and found a highly homologous sequence with 86% identity, which included both coding and adjacent intron regions that included the splice site (Figure 3). We designated this sequence as the rat exon 11. The rat exon 11 was located at about 21 kb upstream of exon 1 in the rat OPRM1 locus of chromosome 1, a distance similar to the 30 kb seen in the mouse OPRM1 gene [24] and the 28 kb in the human OPRM1 gene [28]. However, the sequence of the rat exon 11 predicted only seven amino acids before encountering a stop codon. To isolate potential rat exon 11-associated splice variants homologous to those identified in the mouse OPRM1 gene, we performed RT-PCR using sense primers designed from the rat exon 11 sequence together with two antisense primers from the 3'UTR of exon 4. We identified seven exon 11-associated splice variants, rMOR-1G1, rMOR-1G2, rMOR-1H1, rMOR-1H2, rMOR-1i1, rMOR-1i2 and rMOR-1i3, from rat brain (Figures 1B, 2 &4).

The rat exon 11 contained an alternative splice site that divided the exon into two parts, a pattern similar to that seen in the human exon 11 [28], which we assigned as exon 11a and exon 11b. Alternative usage of the two splice sites in the exons 11a and 11b led to the series of splice variants. rMOR-1G1 contained exons 11a/11b/2/3/4. If the AUG in exon 11a was used, rMOR-1G1 only predicted a peptide with seven amino acids since the stop codon predicted from exon 11b terminated its translation. However, rMOR-1G1 still could use the first AUG from exon 2 as the translational start codon to yield a 6 TM protein, a situation similar to that human hMOR-1G1 [28], the mu_3 _receptor [31] and hMOR-1K [33]. rMOR-1G2 had the same exon composition as rMOR-1G1 except that the stop codon in exon 11b was skipped due a downstream splice site of exon 11a. Thus, like both mMOR-1G and hMOR-1G2, translation of rMOR-1G2 can proceed from the exon 11a AUG to encode a 6 TM protein since the exon 11a reading-frame was in frame with that of exons 2/3/4 (Figures 2 &3). The two AUGs in exon 11a were in the same reading-frame and separated by four amino acids. We arbitrarily assigned the first AUG as the translational start codon for rMOR-1G2, although it is not clear which AUG is actually used.

The other five variants, rMOR-1H1, rMOR-1H2, rMOR-1i1, rMOR-1i2 and rMOR-1i3, contained exons 11a, 1a, 2, 3 and 4, but with alternative splicing among exons 11a, 11b, 1a, 1b and 1c to generate different transcripts. Despite their differences in exon composition, rMOR-1H1, rMOR-1i1, rMOR-1i2 and rMOR-1i3 all predicted the same protein sequence as the original rMOR-1 when using AUG in exon 1a as translational start codon (Figure 1B, 2 &4). Translation from the AUG of exon 11a predicted a short protein sequence due to early translation termination within exon 11b, exons 1b or 1c. The ability of four different exon11-containing transcripts to encoded the same protein as rMOR-1 protein mimics three mouse exon 11-containing variants, mMOR-1H, mMOR-1I and mMOR-1J [24].

**Figure 5:**
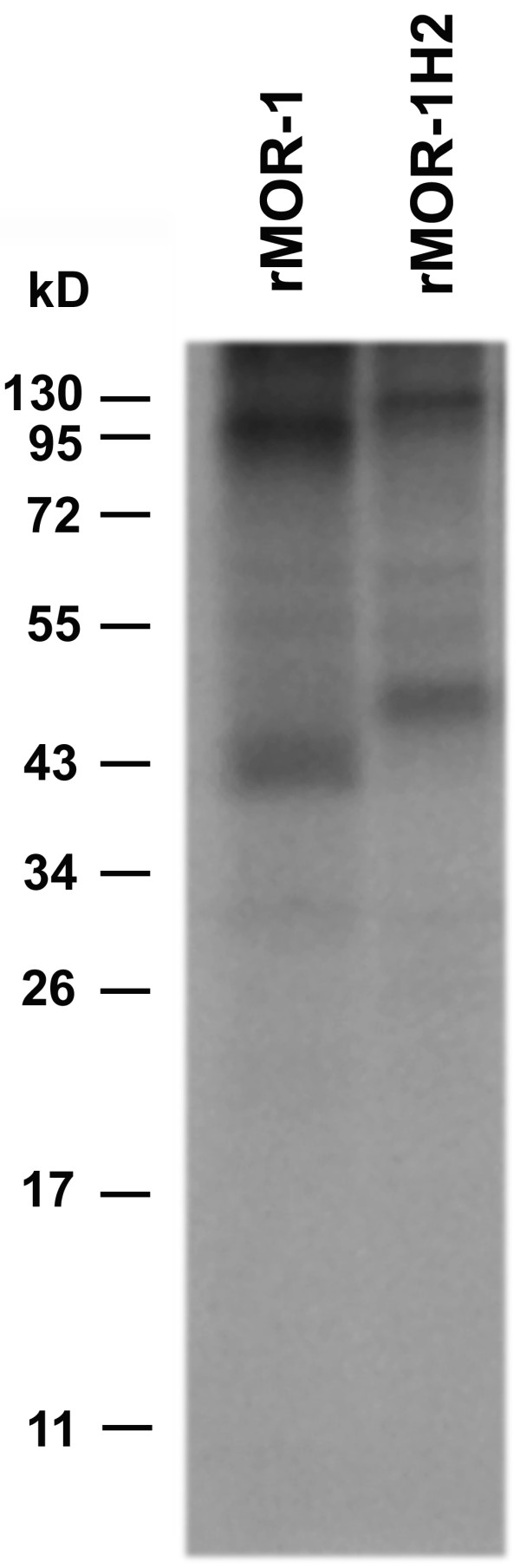
**In vitro translation of rMOR-1 and rMOR-1H2. **In vitro transcription coupled translation was performed as described in the methods section.

The predicted protein sequence of rMOR-1H2 was intriguing. Splicing from exon 11a to exon 1a gave rise to a sequence that predicted an in-frame fusion protein from exon 11a to exons 1a/2/3/4 when the AUG in exon 11a was used as the translational start codon (Figures 2 &4). Thus, rMOR-1H2 encoded a novel receptor protein containing the same amino acid sequence as rMOR-1, but with an additional 50 amino acids at the N-terminus. In vitro transcription coupled translation revealed a molecular weight for rMOR-1H2 that was approximately 5 kD higher than that of rMOR-1, suggesting the preferential usage of the AUG in exon 11a to initiate translation (Figure 5). The 50 amino acid sequence did not contain a predicted transmembrane domain, implying that rMOR-1H2 still encoded a 7 TM protein. Interestingly, the additional sequence did possess a potential N-glycosylation site.

### Expression of the rat exon 11-associated variant mRNAs

**Figure 6:**
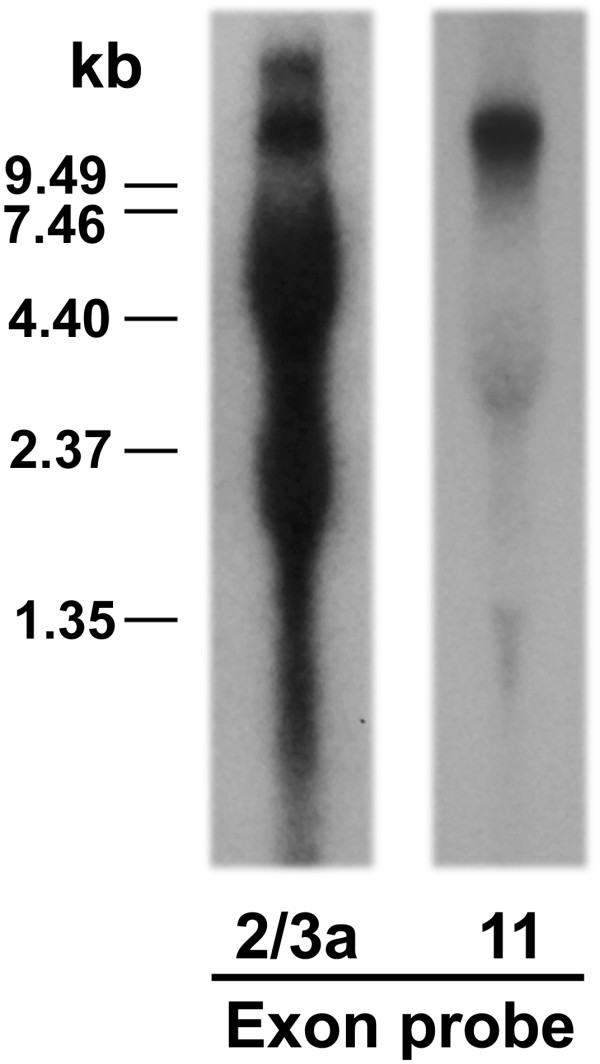
**Northern blot analysis **Northern blots were performed on rat brain using an exon 2/3 probe and an exon 11 probe, as described in the Methods section.

The relative size and abundance of the variant mRNAs was assessed using Northern blot analysis (Figure 6). The exon 2/3a probe, designated to detect most of the variant mRNAs, hybridized several heavy and diffuse bands ranging from 2 - 15 kb, a band pattern similar to Northern blots using mouse and human brains with their respective exon 2/3a probes [24,37]. The exon 11 probe detected a major strong band around 12 kb. A similar band with relatively same size was also seen in the blot with the exon 2/3a probe. Two weaker bands were seen around 1 - 1.5 kb and 2 - 4.5 kb, respectively.

**Figure 7:**
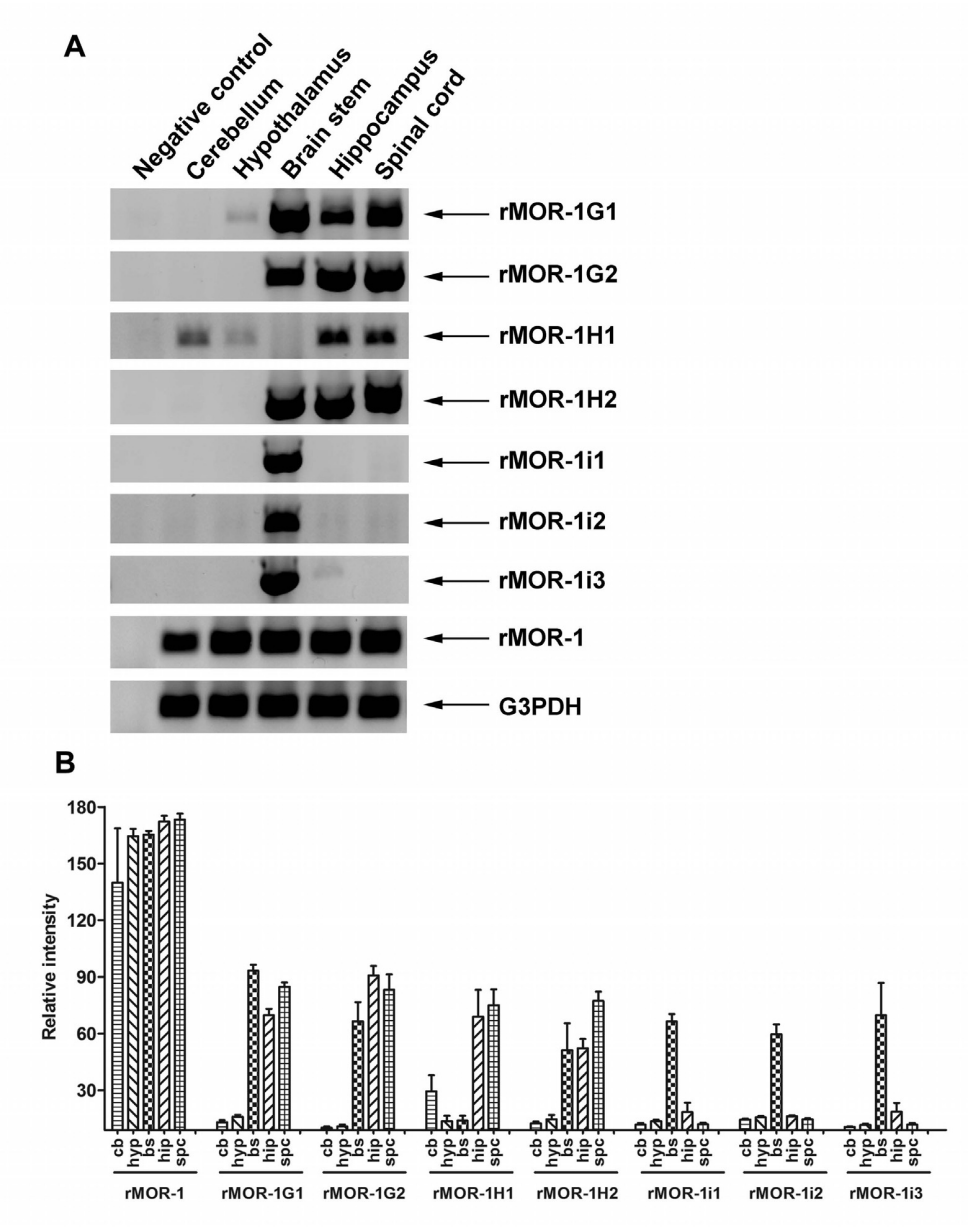
**Regional distribution of the mRNAs from the rat exon 11-associated variants **A. Four sets of total RNAs were extracted from brain regions dissected from four separate groups of rats. Each group contained 1 or 2 rats depending upon the size of the regions. RT-PCRs were performed using primers designed for amplifying rMOR-1G1, rMOR-1G2, rMOR-1H1, rMOR-1H2, rMOR-1i1, rMOR-1i2, rMOR-1i3 and rMOR-1 as described in the Methods. G3PDH was used as RNA loading control. The PCR products were separated on 1% agarose gel, stained with ethidium bromide and photographed using FluorChem 8000 Image System. Only one of four sets data was shown, while the data from other three sets were shown in Additional files 1, 2 &3. B. Quantification of the PCR products from the four sets of RNAs. The band intensities from the agarose gel were quantified with AlphaEase FC software of the Image System and normalized with the band intensities of G3PDH. The data were graphed using GraphPad Prism 4.0 and analyzed with Two-way ANOVA. The results are shown in Table 1.

We next examined the expression of the variant mRNAs in several brain regions using RT-PCR (Figures 7A, 7B, Table 1 & Additional files 1, 2 &3). The rMOR-1 band was observed in all the regions with relatively equal abundance, except for lower levels in the cerebellum. However, the expression of the other mRNAs varied markedly among the regions. rMOR-1G1, rMOR-1G2 and rMOR-1H2 were highly expressed in the brain stem, hippocampus and spinal cord, but had very lower levels in the cerebellum and hypothalamus. In contrast, rMOR-1H1 was abundant in the cerebellum, hippocampus and spinal cord, but limited in the brain stem and hypothalamus. On the other hand, rMOR-1i1, rMOR-1i2 and rMOR-1i3 expression was mainly observed in the brain stem. The brain stem expressed all the variants at relatively high levels except for rMOR-1H1, whereas the hypothalamus expressed the most variants at very low levels. These results suggested region-specific alternative splicing of these variant pre-mRNAs.

**Table 1:** Significance values of the semi-quantitative RT-PCR for the expression of the exon 11 associated variants' mRNAs in the selected brain regions

	cb vs hyp	cb vs bs	cb vs hip	cb vs spc	hyp vs bs	hyp vs hip	hyp vs spc	bs vs hip	bs vs spc	hip vs spc
**rMOR-1**	**ns**	**ns**	**0.05**	**0.05**	**ns**	**ns**	**ns**	**ns**	**ns**	**ns**

**rMOR-1G1**	**ns**	**0.001**	**0.001**	**0.001**	**0.001**	**0.001**	**0.001**	**ns**	**ns**	**ns**

**rMOR-1G2**	**ns**	**0.001**	**0.001**	**0.001**	**0.001**	**0.001**	**0.001**	**ns**	**ns**	**ns**

**rMOR-1H1**	**ns**	**ns**	**0.01**	**0.001**	**ns**	**0.001**	**0.001**	**0.001**	**0.001**	**ns**

**rMOR-1H2**	**ns**	**0.01**	**0.01**	**0.001**	**0.01**	**0.01**	**0.001**	**ns**	**ns**	**ns**

**rMOR-1i1**	**ns**	**0.001**	**ns**	**ns**	**0.001**	**ns**	**ns**	**0.001**	**0.001**	**ns**

**rMOR-1i2**	**ns**	**0.001**	**ns**	**ns**	**0.001**	**ns**	**ns**	**0.00**	**0.001**	**ns**

**rMOR-1i3**	**ns**	**0.001**	**ns**	**ns**	**0.001**	**ns**	**ns**	**0.001**	**0.001**	**ns**

Two-way ANOVA followed by Bonferroni post tests was performed to determine significant difference among the selected brain regions for each variant. cb: cerebellum; hyp: hypothalamus; bs: brainstem; hip: hippocampus; spc: spinal cord; ns: no significance (p > 0.05).

### Characterization of the rat exon 11-associated variants by receptor binding

Of the seven exon 11-associated variants, rMOR-1H1, rMOR-1i1, rMOR-1i2 and rMOR-1i3, predicted the same protein as rMOR-1, while rMOR-1H2 encoded a novel receptor protein with additional 50 amino acids extended at the N-terminal tip of rMOR-1. To examine the pharmacological binding profiles of these variants, we established CHO cell lines stably expressing these variants and examined [^3^H] DAMGO binding. Saturation studies demonstrated similar high affinities of [^3^H] DAMGO for all five variants (Table 2). Although the small difference in *K*_*D *_values between rMOR-1i3 and rMOR-1, rMOR-1H2, rMOR-1i1 and rMOR-1i2 were statistically significant, we believe that these small differences reflected differences in the assays rather than the receptor itself, particularly since they all predict receptors with identical amino acid sequences. While it theoretically might be due to the concurrent generation of the 16 amino acid fragment predicted from the methionine in exon 11a, there is, to date, nothing to indicate that this peptide is actually generated. Competition studies confirmed their mu selectivity (Table 3), with mu ligands such as morphine and M6G potently lowering binding while the kappa_1_-selective opioid U50,488H and the delta-selective ligand DPDPE did not. As expected, the variants with the same predicted protein as rMOR-1 displayed the similar binding characteristics as rMOR-1 itself. rMOR-1H2 bound both agonists and antagonists with affinities indistinguishable from other variants including rMOR-1, indicating that the additional 50 amino acids at N-terminus did not influence opioid binding.

**Table 2:** Saturation studies with [^3^H] DAMGO

Clone	*K*_*D *_(nM)	*B*_*max *_(pmol/mg protein)
rMOR-1	0.39 ± 0.03	0.47 ± 0.07
rMOR-1H1	0.51 ± 0.05	0.40 ± 0.01
rMOR-1H2	0.37 ± 0.02	0.30 ± 0.05
rMOR-1i1	0.36 ± 0.05	0.24 ± 0.02
rMOR-1i2	0.36 ± 0.04	0.26 ± 0.01
rMOR-1i3	0.63 ± 0.06	0.24 ± 0.01

[^3^H] DAMGO binding was performed in membranes of CHO cells stably expressing the indicated variant constructs. The binding parameters were determined by nonlinear regression analysis. Results are the mean ± S.E.M. of at least three independent determinations. P values determined by one-way ANOVA were 0.0013 for *K*_*D *_and 0.0311 for *B*_*max*_. Tukey post hoc analysis determined that rMOR-1i3 was different from rMOR-1, rMOR-1i1, rMOR-1i2 and rMOR-1H2 (p < 0.001), and that there was no significant difference among the variants in *B*_*max*_.

**Table 3:** Competition of [^3^H] DAMGO binding among the rat MOR-1 variants

Ligand	*K*_*i *_Value					
	
	rMOR-1	rMOR-1H1	rMOR-1H2	rMOR-1i1	rMOR-1i2	rMOR-1i3
Morphine	1.5 ± 0.2	2.0 ± 0.3	0.8 ± 0.1	1.3 ± 0.4	1.2 ± 0.2	1.6 ± 0.2
M6G	4.5 ± 0.3	4.5 ± 0.5	2.6 ± 0.6	3.8 ± 0.5	5.1 ± 1.0	6.3 ± 0.7
DADLE	2.4 ± 0.1		1.9 ± 0.3			
DSLET	6.3 ± 0.3		7.2 ± 1.1			
Naloxone	0.8 ± 0.1	0.9 ± 0.1	0.8 ± 0.3	0.9 ± 0.1	0.7 ± 0.1	1.3 ± 0.2
β-Endorphin	2.7 ± 0.2	3.1 ± 0.3	3.8 ± 1.9	3.3 ± 0.3	3.7 ± 0.3	6.3 ± 0.7
Dynorphin A	15.5 ± 0.9	37.5 ± 5.3	21.8 ± 5.1	16.2 ± 3.5	23.6 ± 2.8	34.6 ± 6.8
U50,488H	> 500	> 500	> 500	> 500	> 500	> 500
DPDPE	> 500	> 500	> 500	> 500	> 500	> 500

[^3^H] DAMGO binding was performed in membranes of CHO cells stably expressing the indicated variants. Dissociate constants, *K*_*i *_values, were determined from IC_50 _values of at least three independent determinations. One-way ANOVA was used to compare the *K*_*i *_values for each drug among the variants. Of the opioids, only M6G (p = 0.0157) showed significant difference with lower *K*_*i *_value of rMOR-1H2 as compared to that of rMOR-1i3 (p < 0.01) in Tukey post hoc analysis.

### Functional comparison of rMOR-1 with rMOR-1H2 in agonist-induced [^35^S]GTPγS binding

All five full-length variants with 7 TM contained exon 1. Of these, only rMOR-1H2 encoded a novel protein, differing from the others by the extended N-terminal sequence. Previously, we found that the C-terminal variants of the OPRM1 gene displayed differences in agonist-induced G protein activation despite their small differences in receptor binding profile [25]. To investigate a possible functional effect of the additional N-terminal sequence in rMOR-1H2 on agonist-induced G protein activation, we compared the agonist-induce stimulation profiles of several agonists on [^35^S]GTPγS binding in stably transfected CHO cells expressing rMOR-1H2 and rMOR-1 (Table 4). All the drugs effectively stimulated [^35^S]GTPγS binding. However, we observed differences in both their potencies (EC_50 _value) and efficacies (% maximal stimulation) among the variants. For example, DAMGO and dynorphin A were more potent in rMOR-1H2 than in rMOR-1, as was β-endorphin. Maximal stimulation revealed that morphine was significantly more efficacious in rMOR-1H2 than in rMOR-1. There was little correlation between the EC_50 _and the maximal stimulation, as shown by the fact that dynorphin A was the most efficacious of the ligands tested, despite its lower potency.

**Table 4:** Stimulation of [^35^S]GTPγS binding by opioids in rMOR-1 and rMOR-1H2

	rMOR-1				rMOR-1H2			
	
	**EC**_50_	**EC**_50_/*K*_*i*_	% Max	Relative Efficacy (%)	**EC**_50_	**EC**_50_/*K*_*i*_	% Max	Relative Efficacy (%)
Morphine	59 ± 18	39	180 ± 4	71	70 ± 24	88	225 ± 20*	90

M6G	40 ± 4	9	149 ± 3	59	44 ± 6	17	189 ± 25	75

DAMGO	35 ± 2	90	192 ± 5	76	20 ± 3*	54	209 ± 17	83

β-Endorphin	58 ± 34	21	226 ± 12	89	24 ± 8	6	241 ± 17	96

Dynorphin A	344 ± 64	22	254 ± 6	100	162 ± 12*	7	251 ± 44	100

Membranes were prepared from CHO cells stably transfected with the indicated cDNA constructs and [^35^S]GTPγS binding carried out as described in the Methods section. The maximal stimulation, defined as the percent increase over basal binding, % Max, and the dose of drug needed to elicit 50% of the maximal response, the EC_50_, were calculated by nonlinear regression analysis (GraphPad Prism 4.0). Results are the means ± S.E.M. of at least three independent determinations. Significant differences of the EC_50 _and maximal stimulation between rMOR-1 and rMOR-1H2 were analyzed by Student t-test. Intrinsic activity (EC_50_/*K*_*i*_) was calculated by dividing EC_50 _values by *K*_*i *_values in Table 2. The relative efficacies for the listed opioids were determined for each of the variants, based upon the maximal stimulation values. The drug with the highest level of stimulation for a specific variant was arbitrarily given an efficacy of 100%. Efficacy for all the other compounds for the indicated variant was defined relative to the drug with the greatest maximal stimulation. *: p < 0.05, when compared to rMOR-1.

To obtain a general indication of the intrinsic activity of the various ligands, we compared their EC_50 _values after normalizing for their receptor binding affinity (EC_50_/K_i_). This provides an indication of the receptor occupancy needed to elicit the response. The lower the number, the greater is the intrinsic activity of the ligand. While the EC_50 _values for M6G and DAMGO were similar for rMOR-1, their EC_50_/K_i _ratios differed by approximately 10-fold. A similar situation existed for rMOR-1H2. The potency of dynorphin A in stimulating [^35^S]GTPγS was far less than that of the other ligands, while its EC_50_/K_i _ratio was the lowest, implying the greatest intrinsic activity. The rank order of the EC_50_/Ki values varied from their corresponding rank order of both the EC_50 _and the maximal stimulation values. Comparing the two variants, we also saw different rank-order ratios (Table 4). These results suggested that the extra 50 amino acids influence agonist-induced G protein activation.

## Discussion

Multiple mu opioid receptors were proposed in many years ago, mainly based upon pharmacological studies [3,43-45]. However, to date only a single mu opioid receptor gene has been identified, raising the questions of how a single OPRM1 gene could explain the complex pharmacology of mu opioids in animals and humans. Our early antisense mapping studies suggested different exon combinations for the analgesic actions of the two mu agonists morphine and M6G, raising the possibility of alternative splicing in the OPRM1 gene [17,46]. Since then, much effort has been devoted to identifying these OPRM1variants. To date, over 28 splice variants of the mouse OPRM1 gene have been isolated [22-24,27,35], some of which had been previously identified in humans [21] and rats [20].

The majority of variants were C-terminal variants, differing only at the C-terminal tip. These variants revealed marked differences in their regional distribution at both mRNA and protein level [22-24,27,47-52] and agonist-induced G protein activation and internalization [25,36,37]. We then identified a second set of variants associated with exon 11, a previously unknown exon located 30 kb upstream of exon 1 [24], and established their functional significance in an exon 11 KO mouse model [42]. Disrupting exon 11 diminished M6G and heroin analgesia without affecting morphine or methadone actions, suggesting that exon 11 and its associated variants played an important role in the actions of a subset of mu opioids that include M6G and heroin. A number of C-terminal variants have been isolated from the rat [20,25] and human OPRM1 genes [21,26,37]. We recently isolated a homolog exon 11 and three its associated variants in the human OPRM1 gene [28]. The current studies have now extended a similar splicing pattern to the rat with the identification of a homologous exon 11 and seven associated variants in the rat OPRM1 gene. Additionally, the exon 11 sequence has been predicted from the OPRM1 genomic locus of six other mammalian species through NBCI and Ensembl databases, including chimpanzees, monkeys, guinea pigs, bats, cows and armadillos [53], but not in lower vertebrate species such as fish and amphibians that contain OPRM1 gene.

The nucleotide sequence and genomic location of the rat exon 11 were similar to those in the mouse and human. However, some differences exist between the rat and mouse exons 11. Whereas the rat contains an alternative splice site within exon 11 which splits it into exon 11a and exon 11b, a situation similar to the human exon 11, the mouse does not. Alternative usage of these two splice sites within exon 11, together with a choice of downstream exons, created a number of different variants. The rat exon 11b has a predicted stop codon when translated from its first AUG in exon 11a, leading to only seven amino acids in rMOR-1G1, rMOR-1H1 and rMOR-1i1. However, initiating translation from the first AUG of exon 2 in rMOR-1G1 predicts a 6 TM protein. Using the AUG in exon 1a of rMOR-1H1 and rMOR-1i1, rMOR-1i2, rMOR-1i3 leads to the same protein as the original rMOR-1.

The variants that skip exon 11b can translate through from the AUG in exon 11a, but differed in amino acid sequence depending upon their downstream exons. In rMOR-1i2 and rMOR-1i3, translation using the first AUG in exon 11a still predicted small proteins due to early termination of translation in exons 1b and exon 1c, respectively. However, both rMOR-1i2 and rMOR-1i3 can initiate translation from the AUG of exon 1a to generate the same protein as the original rMOR-1. Thus, together with rMOR-1H1 and rMOR-1i1, a total of four exon 11-associated transcripts can produce the identical rMOR-1 protein, a similar situation seen in the mouse exon 11-aasociated variants. This raises questions regarding why four different splice variants are needed to generate the same protein. It is interesting to speculate, that these differences may differentially regulate their cellular location and their ability to express the protein, but there is no evidence to date to support this possibility.

On the other hand, translation from the AUG of exon 11a also generated the 6 TM protein, rMOR-1G2. Skipping exon 11b maintained the reading frame from AUG of exon 11a through exons 2/3/4. Similarly, skipping exon 11b also enabled rMOR-1H2 to read through, yielding a novel receptor with extra 50 amino acids extended at the N-terminus of rMOR-1, a prediction that was supported by in vitro transcription coupled with translation. Thus, rMOR-1H2 is the first full length (i.e. 7 TM) rat splice variant isolated with a different protein sequence at the N-terminus.

The exon 11 mRNAs are relatively abundant in the brain, as illustrated by Northern blot analysis that displayed a major ~ 12 kb band with intensity comparable to that observed with the exons 2/3 probe. The expression of the exon 11-associated variant mRNAs differed markedly among brain regions, contrasting with the relatively homogenous expression levels of rMOR-1. This suggested that, like the mouse, there is region- and/or cell-specific RNA processing of the variant pre-mRNAs and/or varying levels of upstream promoter activity. Differential expression of the variant mRNAs among brain regions also raised questions regarding their functions. Recently, we observed high correlations between mRNA expression levels, including exon 11-associated variants, in selected brain regions with the degree of morphine and heroin dependence and tolerance among four inbred strains of mice (J Xu, B Kest and YX Pan, unpublished observations). These results suggest a possible contribution of alternative splicing of the OPRM1 gene in mu opioid tolerance and addiction in mice, although the relevance of these correlations needs to be further validated. It will be interesting to see if these correlations also exist in rat.

The genomic location of the rat exon 11 approximately 21 kb upstream of exon 1 suggested the existence of an upstream promoter controlling the expression of the exon 11-associated variants. Preliminary studies indicate that the 5' flanking region of the rat exon 11 has promoter activity, particularly in the neuroblastoma cell lines NIE115 and Be(2)C cells, assessed using a secreted alkaline phosphotase (SEAP) reporter assay (J Xu and XY Pan, unpublished observation).

rMOR-1H2 encoded a full length 7 TM mu opioid receptor with a unique, extended N-terminus. Its similar binding profile is consistent with the other variants was expected since it is believed that the binding pocket is contained within the transmembrane regions, which are identical among all the full length variants. However, the additional N-terminal 50 amino acids in rMOR-1H2 did influence agonist-induced G protein activation, a similar scenario seen in the human N-terminal variant, hMOR-1i [28]. While similar results were observed with the C-terminal variants, but this was more easily understood because of the presumed ability of the C-terminus to influence coupling to transduction proteins. How the additional N-terminal sequence influences receptor activation is as yet unknown.

In the mouse, the exon 11-associated variants mMOR-1G, mMOR-1M and mMOR-1N predict 6 TM variant due to skipping of exon 1, which encodes the first TM. rMOR-1G2 predicted a similar 6 TM protein with translation of exon 11 that resembles mMOR-1G with exon 4 as the last coding exon. While rMOR-1G1 also predicts a 6 TM variant with a terminal exon 4, it requires using the AUG within exon 2 to initiate translation. Despite our efforts, we were unable to isolate rat homologs of the mouse mMOR-1M and mMOR-1N. While it is possible that they do not exist in rats, it also is possible that these homologs are localized to very specific brain regions with a low overall abundance. The functional relevance of the 6 TM mouse variants has been suggested by a range of studies. First, although they do not bind radiolabeled mu agonists with high affinity, the mouse 6 TM variants displayed a moderate binding affinity towards [^3^H]-diprenorphine (K_D _approximately 10 nM; J Xu, GW Pasternak and YX Pan, unpublished observation). Second, the 6 TM variants can physically associate with the regular 7 TM MOR-1 and modulate the expression of the 7 TM receptors on cell surface membrane (J Xu, GW Pasternak and YX Pan, unpublished observation). More importantly, disrupting exon 11 diminished M6G and heroin analgesia without affecting morphine and methadone, suggesting selective roles of the 6 TM exon 11-associated variants in the actions of M6G and heroin. Finally, the conservation of the exon 11 and exon 11-associated variants across species further supports their role.

## Conclusions

We isolated a rat exon 11 and seven exon 11-associated splice variants from the rat OPRM1 gene, resembling splicing in both mice and humans and suggesting conservation of exon 11 and its associated variants in mammals. The rat OPRM1 gene now contains eleven exons spanning over 250 kb and whose combination by alternative splicing generates over sixteen variants. The functional significance of these rat exon 11-associated variants was suggested by the region-specific expression of their mRNAs and the influence of the novel N-terminal sequence on agonist-induced G protein coupling in the N-terminal variant, rMOR-1H2. The existence of the rat exon 11-associated variants raises questions regarding their potential role in mediating the actions of heroin and M6G in rat. The diversity and complexity created by alternative splicing of the rat OPRM1 gene may provide important insights of understanding the diverse responses to the various mu opioids seen in rat.

## Methods

### Genomic database searching

Alignment of the mouse exon 11 sequence in with the rat OPRM1 gene in the Ensembl human genome database revealed a sequence homologous to exon11. The rat exon 11 was mapped approximately 21 kb upstream of exon 1 in the rat OPRM1 locus. There is 86% identity at the nucleotide level between the rat exon 11 and the mouse exon 11 sequences (Figure 3).

### Reverse transcription-polymerase chain reaction (RT-PCR) cloning

Total RNA was isolated from rat brain or selected brain regions by the guanidinium thiocyanate phenol-chloroform extraction method [54] and reverse transcribed with random primers and Superscript II reverse transcriptase (Invitrogen) as previously described [22,55] or with an antisense primer from exon 4 (E4-AN1, 5'-CAT GTG CAG AGT GAA GTA GCC AGA G-3') and Superscript III reverse transcriptase (Invitrogen) following the manufacture's protocol. In a 20 μl of RT reaction with random primers and Superscript II, 5 μg of RNA together with 260 ng of random primer was first incubated at 70°C for 5 min and then quickly cooled on ice for 2 min. Following adding the reaction buffer together with 10 mM DTT and 1 mM dNTP and warming the mixture at 37°C for 2 min, 260 units of Superscript II were added. The mixture with the enzyme was incubated at room temperature for 10 min, then at 37°C for 5 min and finally at 42°C for 90 min. The reaction was terminated by heating at 75°C for 15 min. In a 20 μl of RT reaction with E4-AN1 primer and Superscript III, 5 μg of RNA together with 10 pmol of E4-AN1 primer was first incubated at 70°C for 5 min and then quickly cooled on ice. The reaction buffer together with 5 mM DTT, 1 mM dNTP and 200 units of Superscript III was added. The reaction was incubated at 53°C for 90 min and terminated by heating at 75°C for 15 min. Two-step or nested PCRs was used to amplify exon 11-associated full-length clones using Platinum Taq DNA polymerase (Invitrogen). The first-step PCRs were carried out using 5 μl of RT reaction as template with the appropriate primers (see below) for 39 cycles after 2 min at 94°C, each cycle consisting of a 20 sec denaturing step at 94°C, a 20 sec annealing step at 65°C and a 2 min extension at 72°C. In the second-step PCRs, 2 μl of the first-step PCR products was used as template with appropriate primers (see below) using the same PCR cycling conditions as the first-step PCR. The primers used for rMOR-1G1, rMOR-1G2, and rMOR-1H2 were: two sense primers from exon 11 sequence obtained from the genomic alignment (E11-SE1: 5'-CTT CCC ATA AGT CAT TTG CTG TCC TTG-3' and E11-SE2: 5'-GAA GAG GAA CAC CGA AAC TGG GAA GC-3') and two antisense primers from exon 4 (E4-AN1 and E4-AN2: 5'-GAC AGC AAC CTG ATT CCA CGT AGA TG-3'); for rMOR-1H1 and rMOR-1i1, an exon 11 sense primer (E11-SE3: 5'-GAA GGA TGG GAT CTG GTC CAA TGC TGT AAG CTT TCT CCA AGT CCG CAT TCC AAA AAC TGG ACA GGG AGA TAG AAA TCA AGA GGG GAA GTT ACC TCA G-3') and the two exon 4 antisense primers (E4-AN1 and E4-AN2); for rMOR-1i2, a exon 11 sense primer (E11-SE4: 5'-GAA GGA TGG GAT CTG GTC CAA TGC TTG CAT G-3') and E4-AN1 primer; and for rMOR-1i3, an exon 11 sense primer (E11-SE5: 5'-GCT TGA AGG ATG GGA TCT GGT CCA ATG CTA TAC GAG-3') and E4-AN1 and E4-AN2 primers. All PCR fragments were subcloned into pcDNA3.1/V5His-TOPO vector (Invitrogen) and sequenced with appropriate primers in both orientations.

### Northern blot analysis

Northern blot analysis was performed as described [22,55]. Briefly, 20 μg of total brain RNA/lane was separated on a 0.8% formaldehyde agarose gel, and transferred to GenePlus membrane. The membranes were hybridized with either a 257 bp ^32^P-labeled exon 11 probe generated by PCR with a sense primer (E11-SE1) and an antisense primer (E11-AN1: 5'-GAG GTA ACT TCC CCT CTT GAT TTC TAT CTC CC-3') from exon 11 or a 685 bp ^32^P-labeled exons 2 & 3a probe by PCR with a sense primer from exon 2 (E2-SE1: 5'-GAC TGC CAC CAA CAT CTA CAT TTT CAA C-3') and an antisense primer from exon 3 (E3-AN1: 5'-GTT CGT GTA ACC CAA AGC AAT GC-3').

### Regional expression of the variant mRNAs

The selected brain regions were dissected from four separate groups of rats. Each group had one or two rats depending upon the size of the region. Total RNAs extracted from the selected brain regions were reverse-transcribed with an E4-AN1 primer and Superscript III as described in RT-PCR cloning (see above). The first-strand cDNAs were used as templates for two-step or nested PCRs. For exon 11-associated splice variants, the first-step PCRs were performed using 5 μl of RT reaction as template and a sense primer from exon 11 (E11-SE1) and an antisense primer from exon 4 (E4-AN1) for 35 cycles with the same PCR cycling conditions as described in RT-PCR cloning. In the second-step PCRs, 3 μl of the first-step PCR products was used as template with appropriate primers (see below). The primers used in the second-step PCRs were designed to specifically amplify each variant and listed as following: for rMOR-1G1, a sense primer (G1-SE: 5'-GAA GTT ACC TCA GAT ACA CCA AAA TGA-3') and an exon 3 antisense primer (E3-AN2: CAG CAG ACG ATA AAT ACA GCC ACG-3'); for rMOR-1G2, a sense primer (G2-SE: 5'-GGT CCA ATG CTA TAC ACC AAA ATG-3') and E3-AN2 primer; for rMOR-1H1, a sense primer (H1-SE: 5'-AAG TTA CCT CAG GGC TGG TCC-3') and an exon 2 antisense primer (E2-AN1: 5'-ATG TTC CCA TCA GGT AGT TGA CAC TC-3'); for rMOR-1H2, a sense primer (H2-SE: 5'-GGT CCA ATG CTG GCT GGT CC-3') and E2-AN1 primer; for rMOR-1i1, a sense primer (I1-SE: 5'-AAG TTA CCT CAG TGC ATG GAG ACC-3') and E2-AN1 primer; for rMOR-1i2, a sense primer (I2-SE: GGT CCA ATG CTT GCA TGG AGA C-3') and E2-AN1 primer; for rMOR-1i3, a sense primer (I3-SE: 5'-GGT CCA ATG CTA TAC GCG GA-3') and E2-AN1 primer. For detecting rMOR-1, the first-step PCRs were performed using the same PCR cycling conditions with 5 μl of RT reaction, an exon 1c sense primer (E1-SE1: 5'-CCC ACT TTA CAC TCG TTT ACA CGG-3') and E4-AN1 primer. In the second-step PCRs, 3 μl of the first-step PCR products was used as template with an exon 1 sense primer (E1-SE2: 5'-GAC AGC CTG TGC CCT CAG ACC-3') and E2-AN1 primer. The second-step PCRs were carried out for 35 cycles after 2 min at 94°C, each cycle consisting of a 20 sec denaturing step at 94°C, a 20 sec annealing step at 60 - 65°C and 45 - 90 sec extension at 72°C, depending upon melting temperature of primers and length of amplicons. The lengths of the PCR products were consistent with their predicted sizes: 606 bp for rMOR-1G1, 604 bp for rMOR-1G2, 539 bp for rMOR-1H1, 537 bp for rMOR-1H2, 794 bp for rMOR-1i1, 793 bp for rMOR-1i2, 1051 bp for rMOR-1i3 and 217 bp for rMOR-1. The sequences of the PCR products were confirmed by sequencing with appropriate primers. A negative control using ddH_2_O as template was included for each variant throughout the two-step PCRs. RNA loading was estimated by parallel one-step PCRs with a pair of primers for glyceraldehydes 3-phosphate dehydrogenase (G3PDH) (Clontech). The PCR products were separated on 1% agarose gel, stained with ethidium bromide. The agarose gel was photographed and analyzed using a FluorChem 8000 Image System (Alpha Innotech).

### In vitro transcription coupled translation

The full-length cDNAs of rMOR-1 and rMOR-1H2 in the pcDNA3.1/V5His-TOPO vector were transcribed and translated in vitro with a TnT T7 coupled reticulocyte lysate system (Promega) following the manufacturer's protocol. Briefly, the plasmids were incubated with T7 RNA polymerase and reticulocyte lysate in the presence of 0.04 mCi of [^35^S]methionine (> 1000 Ci/mmol; PerkimElmer) at 25°C for 90 min. The translated products were separated on a 12% SDS-polyacrylamide gel, and the gel was treated with Amplify (GE Life), dried and exposed to Kodak BioMax MR film.

### Expression of rMOR-1H1, rMOR-1H2, rMOR-1i1, rMOR-1i2 and rMOR-1i3 in Chinese hamster ovary (CHO) cells

The rMOR-1H1/pcDNA3.1-TOTO, rMOR-1H2/pcDNA3.1-TOPO, rMOR-1i1/pcDNA3.1-TOPO, rMOR-1i2/pcDNA3.1-TOPO, rMOR-1i3/pcDNA3.1-TOPO and rMOR-1/pcDNA3.1(-) plasmids were used to transfect Chinese Hamster Ovary (CHO) cells by LipofectAMINE reagent (Invitrogen). Stable transformants were obtained 10 - 14 days after selection with G418 and screened with a [^3^H]DAMGO binding assay.

### Receptor binding assays

Membranes were prepared from stable transfectants as described previously [22]. Saturation and competition binding assays were performed with [^3^H]DAMGO at 25°C for 60 min in 50 mM potassium phosphate buffer, pH 7.4, containing 5 mM magnesium sulfate. Specific binding was defined as the difference between total binding and non-specific binding, determined in the presence of 10 μM levallorphan. *K*_D _and *K*_i _values were calculated by non-linear regression analysis (GraphPad Prism 4.0, Carlsbad, CA). Protein concentrations were determined using the Lowry method as previously described using bovine serum albumin (BSA) as the standard [22,28].

### [^35^S]GTPγS binding assay

Membranes prepared from stable transfectants were incubated in the presence and absence of indicated opioids for 60 min at 30°C in the assay buffer (50 mM Tris-HCl, pH 7.7, 3 mM MgCl_2_, 0.2 mM EGTA, 10 mM NaCl) containing 0.05 nM [^35^S]GTPγS (> 1000 Ci/mmol, PerkimElmer) and 60 μM GDP, as previously reported [25,36,37]. Basal binding was determined in the presence of GDP and absence of drug. The reaction was terminated by rapid filtration under vacuum through glass fiber filters, followed by three washes with 3 ml of ice-cold 50 mM Tris-HCl, pH 7.4. Bound radioactivity was measured by liquid scintillation spectrophotometry in Liquid Scintillation Analyzer (TRI-CARB 2900TR, PerkimElmer) after overnight extraction in 5 ml liquiscint scintillation fluid (National Diagnostic Inc.).

## List of abbreviations

M6G: morphine-6β-glucuronide; DAMGO: [_D_-Ala^2^,*N*-MePhe^4^,Gly-ol^5^]enkephalin; MOR: mu opioid receptor; OPRM1: mu opioid receptor gene; RT: Reverse-transcription; PCR: polymerase chain reaction; KO: knockout;

## Competing interests

The authors declare that they have no competing interests.

## Authors' contributions

JX and MX performed RT-PCR, Northern blot analysis, in vitro transcription coupled translation, receptor binding and [^35^S]GTPγS binding assays. GCR dissected brain regions for RNA extraction. Y-XP designed and coordinated the experiments and analyzed data. Y-XP and GWP oversaw the project and wrote the manuscript. All authors read and approved the manuscript.

## Supplementary Material

Additional file 1 **Regional distribution of the mRNAs from the rat exon 11-associated variants (repeated experiment 1) **Figure S1. All the procedures were performed with a separated group of rat as described in the Methods section and Figure 7 legend. Click here for file

Additional file 2 **Regional distribution of the mRNAs from the rat exon 11-associated variants (repeated experiment 2) **Figure S2. All the procedures were performed with a separated group of rat as described in the Methods section and Figure 7 legend. Click here for file

Additional file 3 **Regional distribution of the mRNAs from the rat exon 11-associated variants (repeated experiment 3) **Figure S3. All the procedures were performed with a separated group of rat as described in the Methods section and Figure 7 legend. Click here for file
