# Supplementary figures and images for: Identification and characterization of seven new exon 11-associated splice variants of the rat mu opioid receptor gene, OPRM1
Source: Mol Pain. 2011 Jan 21;7:9. doi: 10.1186/1744-8069-7-9 (PMC3057186; doi:10.1186/1744-8069-7-9)

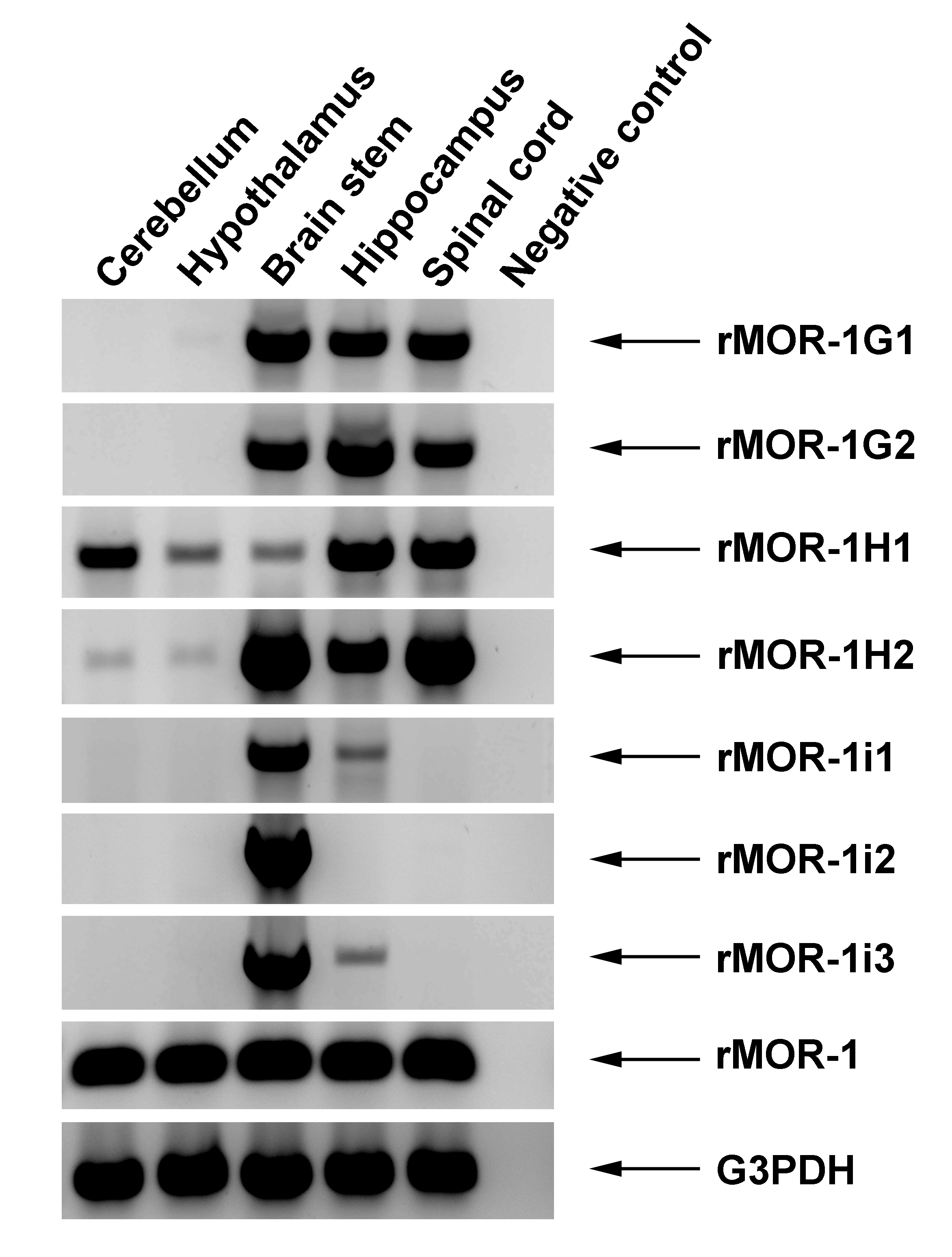

Supplement: Additional file 1 — Regional distribution of the mRNAs from the rat exon 11-associated variants (repeated experiment 1) Figure S1. All the procedures were performed with a separated group of rat as described in the Methods section and Figure 7 legend. [file 1744-8069-7-9-S1.TIFF]

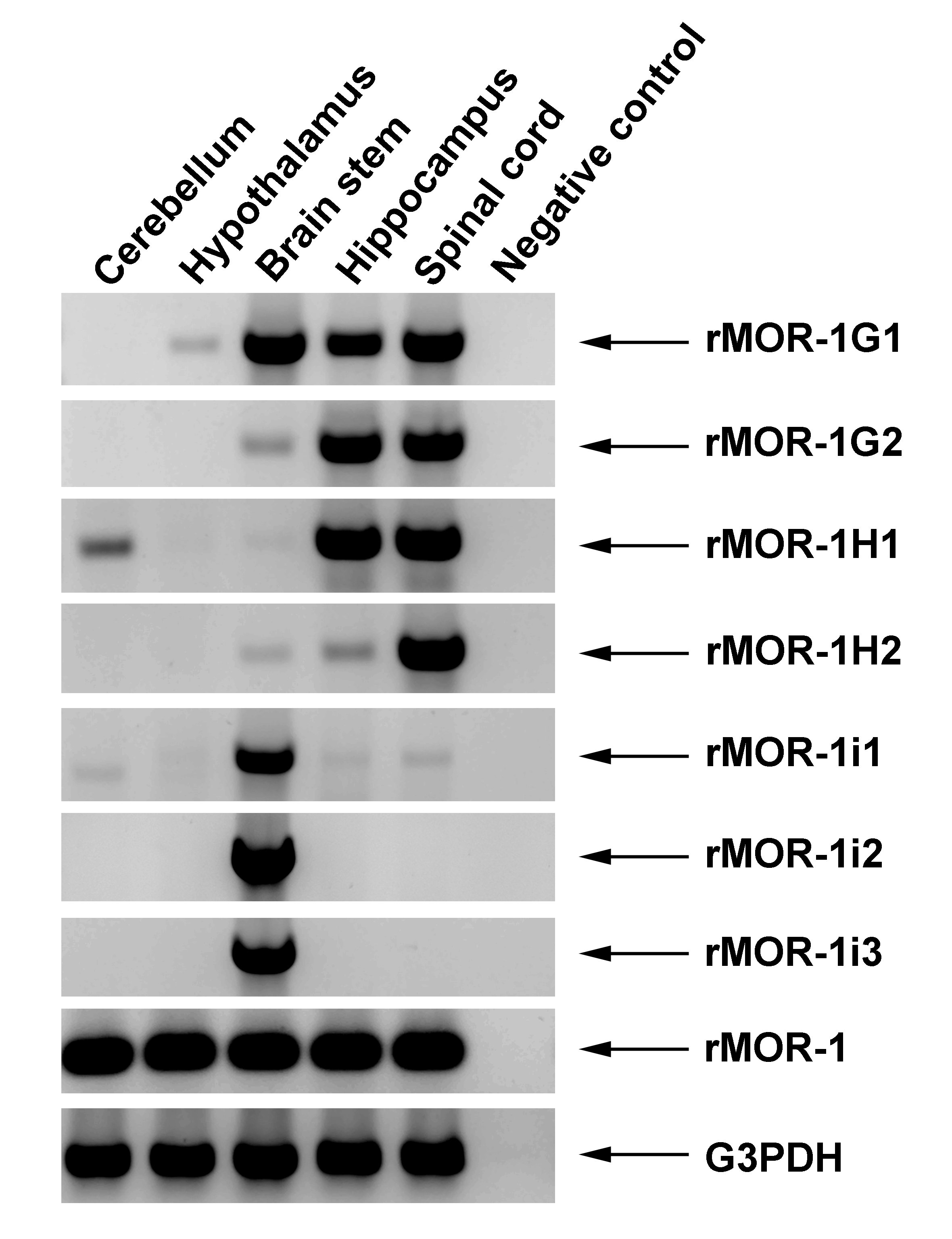

Supplement: Additional file 2 — Regional distribution of the mRNAs from the rat exon 11-associated variants (repeated experiment 2) Figure S2. All the procedures were performed with a separated group of rat as described in the Methods section and Figure 7 legend. [file 1744-8069-7-9-S2.TIFF]

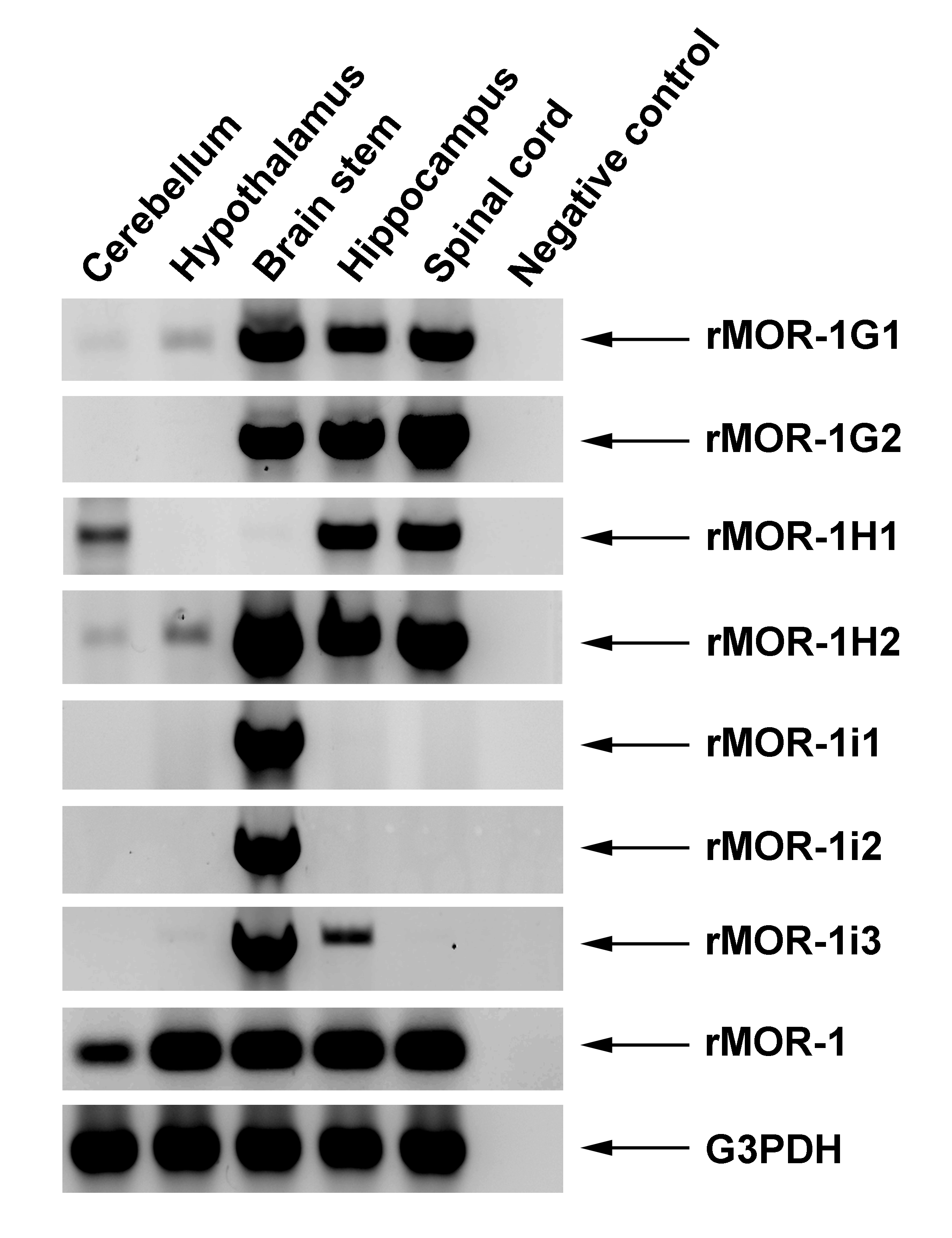

Supplement: Additional file 3 — Regional distribution of the mRNAs from the rat exon 11-associated variants (repeated experiment 3) Figure S3. All the procedures were performed with a separated group of rat as described in the Methods section and Figure 7 legend. [file 1744-8069-7-9-S3.TIFF]
